# Assessing Phase-Change Materials as Effective Long-Term Biosensors in Limb Prosthetics

**DOI:** 10.3390/bios13100944

**Published:** 2023-10-22

**Authors:** Robert Johnston, Danielle Sell, Goeran Fiedler, Anita Singh

**Affiliations:** 1 Biomedical Engineering, Widener University, Chester, PA 19013, USA; 2 Prosthetics and Orthotics, University of Pittsburgh, Pittsburgh, PA 15219, USA; 3 Bioengineering Department, Temple University, Philadelphia, PA 19122, USA

**Keywords:** phase-change materials, biosensors, prosthetic liner, activity levels, gait

## Abstract

Monitoring and controlling the microclimate at the skin–socket interface of limb prostheses is an important, yet unresolved, clinical problem. Phase-change materials (PCMs) represent a promising biosensor technology that holds the potential to both detect and alter (i.e., stabilize) changes in the temperature of a hybrid biological/mechanical system, such as a prosthesis. The biologically inspired sensor capabilities of PCMs can enhance the internal socket conditions and offer improved comfort and suspension while minimizing skin injuries for prosthesis users. This study investigated how prosthetic liners equipped with PCM biosensors affected the long-term outcomes for prosthesis users. In this double-blinded longitudinal crossover study, a cohort of transtibial prosthesis users wore regular conventional liners for six months and PCM liners for another six months. Prosthesis utilization, physical performance, and gait symmetry were studied using Modus StepWatch, the 2-minute walk test, and the TekScan F-Scan gait test, respectively. Measured parameters from these various tests, acquired at multiple timepoints during the study, were compared pairwise between the two liners per individual. While the obtained quantitative data trends, such as the gait symmetry, favored the PCM liners, no statistically significant differences were found between the PCM and conventional gel liners in any of the study parameters.

## 1. Introduction

A prosthetic limb is commonly prescribed after major limb loss, which may result from diseases such as diabetes, traumatic accidents, or other causes [1]. Limb loss is a significant, life-changing event, and while prosthesis utilization offers mobility and a better quality of life, it does lead to alterations in normal gait and increases the risks of new injuries that affect overall health [2]. Users of limb prostheses are at an increased risk of pressure sores, impaired blood perfusion, and injuries from accidental falls [3,4,5]. Many of these issues have their origin at the interface between the residual limb and the prosthetic socket, where prosthesis function conflicts with user comfort (specifically, increased residual limb temperatures) [6].

Sweating and heat buildup occur in 57% of prosthesis users, posing significant secondary complications [7]. The fitting and structural stability requirements of liner and socket systems can entail poor thermal conductivity, which is the main cause of increased residual limb temperatures, leading to excess sweating [8]. Prosthesis users who experience excess sweating wear their prostheses less, which may lead to lower activity levels, thus decreasing user satisfaction and health-related quality of life [9]. There is a need to investigate technologies that can be used in the fabrication of prosthetics to better control the thermal properties and perspiration of the residual limb.

Limited research has been conducted to control the temperature environment between the residual limb and the liner [10,11]. A study by McGrath et al. in 2019 compared two perforated liners. The first liner, the Silcare Breathe Cushion, utilized an elevated vacuum suspension with perforations along the length of the liner and a distal one-way valve to allow for suction suspension. The second, the Silcare Breathe Locking, also utilized a vacuum suspension system with perforations along the length of the liner, but it additionally included a valve on the distal portion that opened and closed during loading and unloading cycles. This created a vacuum pressure that pulled air through the liner perforations. The results from three participants showed substantial improvements when wearing these liners, but since perforations pose the risk of having areas with high stress concentrations, they increase the chances of damaging the liner or the skin along those regions [10]. Other studies have investigated thermoregulatory systems that use a fan and an aluminum-enclosed heat-sink pump to increase or decrease the temperature within the prosthetic socket [11,12,13]. The results showed promise in controlling the temperature inside and outside the liner at various distances from the thermal pump in a short time, but clinical testing is currently unavailable to determine its feasibility.

Phase-change material (PCM) technology in prosthesis liners has been reported to improve temperature control and, consequently, reduce sweating in users [11,14,15]. PCMs are thermoresponsive biosensors that react to ambient temperature changes by transitioning between solid and liquid phases at a designated threshold, thereby steadying the temperature of the material. This principle has been proposed to be employed in PCM liners that absorb the heat built in a residual limb, thereby preventing the development of perspiration. It further stabilizes the skin temperature by releasing stored heat when the body cools, keeping the user comfortable [11]. A double-blind randomized study by Wernke (2015) compared PCM liners to conventional gel liners. The study had 16 transtibial prosthesis users wear the conventional liner and the PCM-based “SmartTemp” liner while riding a stationary bike for 25 min. Four thermocouples were placed on the medial anterior, medial posterior, lateral posterior, and lateral anterior residual limb regions. The users’ sweat was measured by weighing a towel used for wiping the residual limb and the inner surface of the test liner before and after the exercise to calculate the accumulated sweat. The results indicated that the socket temperature and perspiration inside the socket stayed lower and rose slower in PCM-based liners than in conventional gel liners [11]. This study supports the use of PCM-based liners. However, being a short, lab-based study, its clinical significance is limited, and long-term use studies are warranted to further understand the efficacy of PCM liners in prosthesis users.

The goal of this study was to fill the critical gap in understanding of the short-term and long-term benefits of PCM liners when compared to conventional liners. This study investigated the short- and long-term effects of PCM liners on users’ activity levels, performance, and gait biomechanics when compared to conventional liners. In addition to hypothesizing that wearing PCM liners would increase users’ activity and performance levels [16], we also hypothesized that the use of PCM liners, when compared to conventional liners, would result in a more symmetrical (between legs) gait, as measured by the total ground reaction force, stride time, and maximum peak pressure distribution in the foot. Furthermore, we postulate that the improvements in activity levels, performance, and gait symmetry when using PCM biosensor technology in prosthetic liners indicate an improvement in the prosthesis performance and utilization.

## 2. Materials and Methods

The study protocol was reviewed and approved by the participating organizations’ institutional review boards.

### 2.1. Inclusion/Exclusion Criteria

The study participants were recruited and screened by certified prosthetists according to the following criteria: Inclusion criteria were the use of a prosthesis with liner suspension, at least one year of prosthesis use, a well-fitting socket, a matured residual limb (i.e., stable limb volume) that had not required socket modifications in the past six months, the ability to walk with the prosthesis outdoors without notable limitations (i.e., K-Level 3 or higher), stable body weight, and absence of acute medical conditions that would temporarily affect the ability to use prostheses. Notable exclusion criteria included the use of a non-standard liner size, current use of a PCM liner as the regular suspension system, known allergies to liner materials, and any inability to understand the protocol or to comply with the associated tasks, such as keeping track of days without using the liner and rotating between each pair to provide similar wear to each liner. These criteria, along with consent to participate and be recorded for various parts of the study, were written in a participant consent form and signed by all participants and researchers prior to beginning the study.

### 2.2. Initial Screening

A study intake form was filled out by the prosthetist to correctly order the study liners for each participant. Measurements were taken at the residual limb’s distal and proximal ends. The suspension type, whether cushion or locking, size, material, and a uniform or progressive profile were noted. These details were used to order a total of four study liners: two PCM liners (“SmartTemp”, Ohio Willow Wood Company, Sterling, OH, USA), and two liners made from a conventional silicone gel material, representing the control condition, which were custom-made to look identical to the intervention liners. Two of the same liners were given out at a given timepoint to allow for daily switching and cleaning, consistent with established clinical guidelines.

### 2.3. Randomization

All four liners (two PCM and two conventional) per participant had the same appearance and imprinted wording, but different serial numbers. Randomization was performed using proc survey select, available in SAS version 9.4. The process used a block randomization scheme with block lengths of 2 and 4. This ensured a balanced distribution between the groups, while minimizing any chance of guessing which participant would start with the test liner. Group 1 participants started with the PCM liner, while Group 2 participants started with the conventional liner. This characterization, however, was blinded to all parties other than the biostatistician. Once the liners were ordered and received, the participants were fitted with their study liners. An activity monitor (StepWatch, Modus Health, Washington, DC, USA) was affixed to their prosthetic leg by a Velcro strap.

### 2.4. Study Design and Data Collection

**Figure 1:**
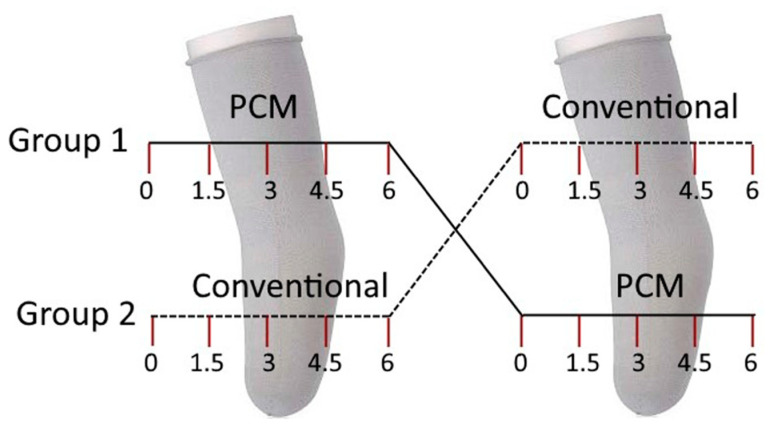
Cross-sectional study details; given timepoints are in months.

Step counts were acquired every two weeks, and the 2-minute walk test (2MWT) and gait tests, using F-Scan (TekScan, Norwood, MA, USA) sensors for ground reaction force data collection, were administered every 6 weeks. The total duration of the study was one year (12 months), with six months in each liner group: conventional or PCM (Figure 1).

#### 2.4.1. Two-Week Follow-Up

The daily step count was acquired using the StepWatch, which was affixed to the prosthetic leg by a Velcro strap. In addition to reading out daily step count data during the two-weekly follow-up sessions, the participants were also asked about liner performance and days on which the liners were not used, if any. After the readout, the StepWatch was reset to empty the data logger for the next 2-week data collection period.

#### 2.4.2. Six-Week Follow-Up

At the six-week site follow-ups, in addition to reading out daily step count data, a 2-minute walk test (2MWT) and an F-Scan gait test were administered. The two provided liners (from the same liner group) were also inspected by a clinical healthcare provider during this visit to confirm that the liners were maintaining their properties.

#### 2.4.3. MWT

For the 2MWT, cones were placed 5 m apart, with a 1 m turn zone at each cone. The participants were asked to walk at a comfortable pace for 2 min while the research clinician followed behind with a measuring wheel (Komelon ML1212 Meter-Man 4-inch). The distance wheel recorded the distance walked by the participant, in feet. The participants were also asked to remain silent, to walk in a loop rather than a figure of 8, and were informed that they could stop at any point if necessary. Walkers or assistance devices were permitted if necessary.

F-Scan gait test: F-Scan is an ultrathin, in-shoe sensor that captures timing and pressure information for foot function and gait analysis. These shoe inserts, which are lined with pressure sensors (960 different pressure-sensing locations (sensels) and a spatial resolution of 4 sensels/cm^2^), were placed in the participants’ shoes during each six-week follow-up timepoint. Height and weight in newtons were used to calibrate the device for each participant. The participants were asked to stand up and shift their weight to ensure that the calibration was successful. The participants were then asked to walk for 10 s until the device stopped recording. Using the F-Scan software (V10.0), the ground reaction force (N), stride time (s), and foot (fore, mid, and rear) pressures (kPa) were recorded and analyzed. Gait data were also analyzed to determine symmetry between the left and right limbs. Gait symmetry was used as an indicator of functional differences in the contribution of each limb during walking. The symmetry index (SI) was calculated using Equation (1). By taking the difference between the left and right limbs and dividing that by the average value between the left and right limbs, a percentage assessment can be evaluated. For analysis, SI = 0 indicates full symmetry, while SI ≥ 100% indicates an asymmetrical gait.

(1) $$SI=\frac{\left|X_{L}-X_{R}\right|}{0.5*(X_{L}+X_{R})}*100\%\;$$

**Equation (1)**: symmetry index.

#### 2.4.4. Six-Month Appointment

At the six-month appointment, after acquiring the StepWatch recording and completing the 2MWT and F-Scan gait test, the participants turned in their two study liners and received the two new liners. All data collection steps followed as previously described.

#### 2.4.5. 12-Month Appointment

The 12-month appointment marked the end of this study. After performing the StepWatch recording and completing the 2MWT and F-Scan gait test, the participants were asked which liner pair they preferred, and they were given all four liners for continued use if so desired.

### 2.5. Statistical Analysis

Data analysis was performed by members of the research team who were blinded to the participant groups. Descriptive statistics was conducted, as well as parametric (*t*-tests) and non-parametric comparisons of the outcome variables, including the “Days without prosthesis use” (defined as a step count lower than 10% of the individual’s daily average), “2-min walk distance”, and “F-Scan parameters” across the two liner conditions at the end of each intervention period. A significance criterion of *p* = 0.05 was applied.

## 3. Results

Fourteen participants agreed to participate in the study (Table 1). The participants in the included sample were on average 58 years old (standard deviation (SD) 10 years), with a height of 176.5 cm (SD 4.5 cm) and a body weight of 94 kg (SD 27 kg). At the beginning of the study, they had been living with limb loss for an average of eleven years (SD 16 years), and their mobility rating, measured by the PLUS-M survey [17], was on average in the 59th percentile (range: 48th to 67th percentile) of the overall lower limb loss population.

### 3.1. Activity Level: Step Counts

**Figure 2:**
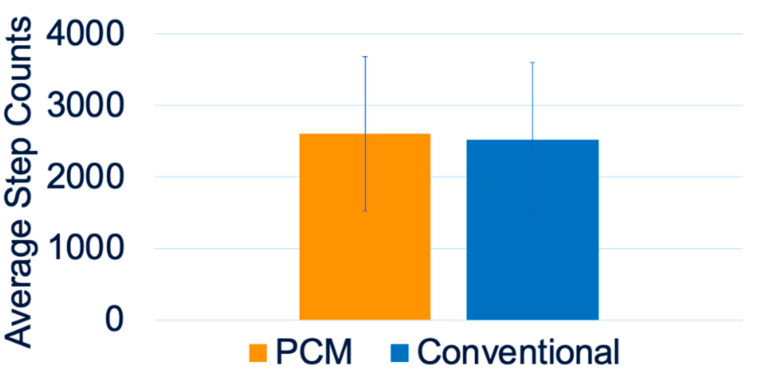
Average daily counts for the conventional and PCM groups.

**Figure 3:**
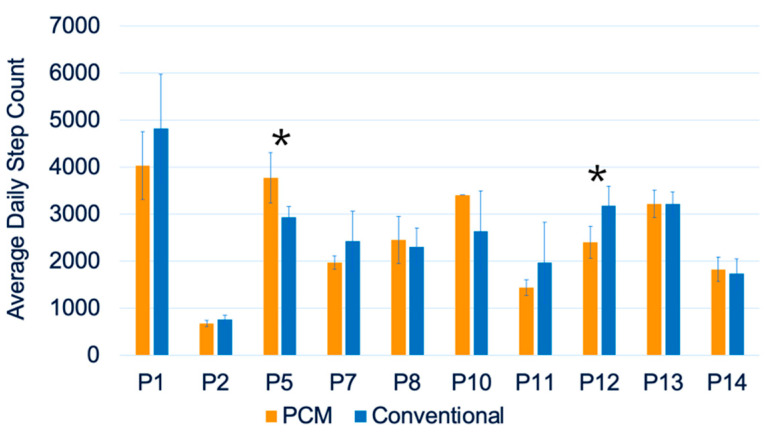
Average daily step counts per participant; * *p* < 0.05.

**Figure 4:**
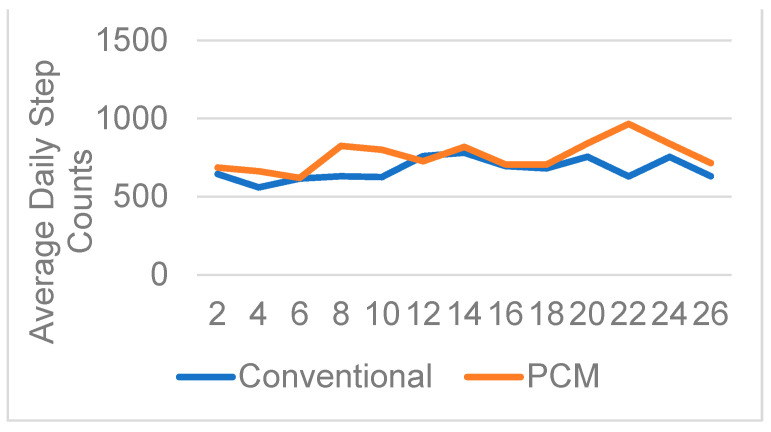
Average daily step counts for participant #2 over 26 weeks.

The average daily step counts acquired using the StepWatch for the two groups are summarized in Figure 2. Independent *t*-tests for average daily step counts showed no significant differences between the PCM liners and the conventional liners (Figure 2). Figure 3 summarizes the step count comparison between the two liners—namely, PCM and conventional—for each participant, analyzed in biweekly intervals over 26 weeks for each liner. A paired *t*-test for each participant’s biweekly average daily step counts showed significant differences in participants #5 and #12 (Figure 3). Figure 4 shows the trend of prosthetic utilization over the 26-week period, with no significant change in step count at any timepoint, including the baseline for participant #2. A similar trend of no significant differences was observed between the PCM and conventional liners at all study timepoints for all fourteen participants.

### 3.2. Performance

**Figure 5:**
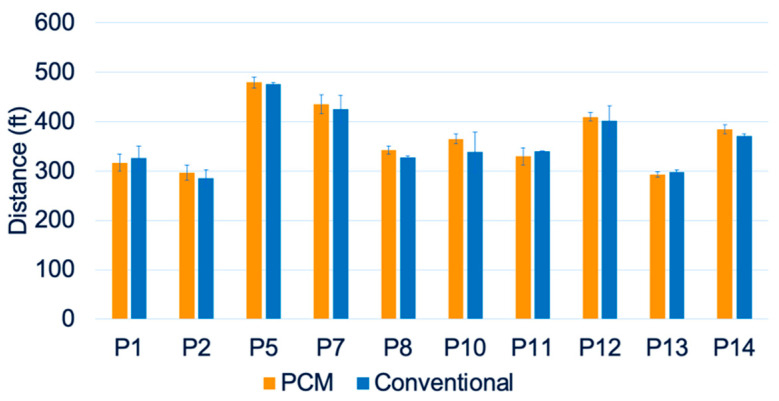
Average 2MWT distances for all participants.

**Figure 6:**
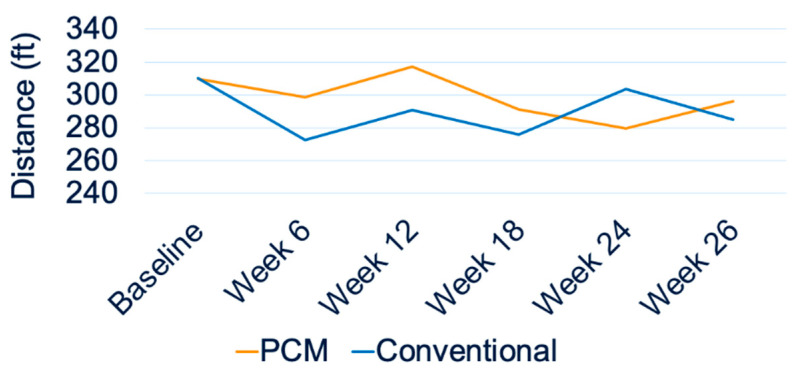
Two-minute walk test (2MWT) distances for participant #2 over 26 weeks.

All fourteen participants completed the 2MWT at each timepoint. Figure 5 shows a comparison of the average 2MWT distances between the PCM and conventional liner groups. Independent *t*-tests and paired *t*-tests for each participant showed no significant differences between the PCM and conventional liner groups in 2MWT distances (Figure 5). Figure 6 shows the trend of prosthetic utilization over the 26-week period, with no significant change in 2MWT distance at any study timepoint, including the baseline for participant #2. A similar lack of significant differences in the 2MWT distance was observed between the two liners at all study timepoints for all fourteen participants.

### 3.3. Gait Analysis: F-Scan

**Figure 7:**
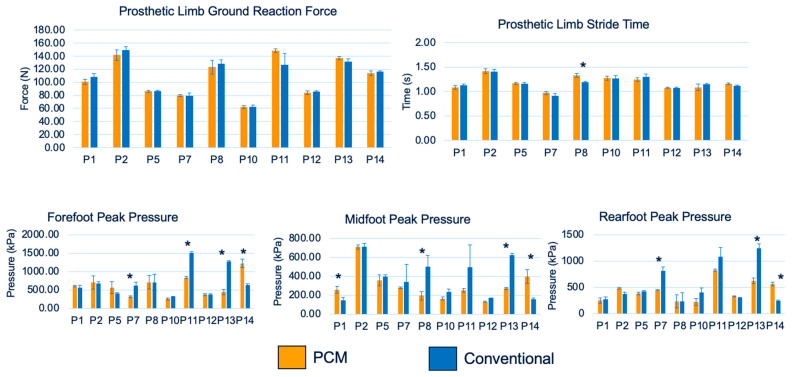
F-Scan data including the average ground reaction force (N), stride time (s), and foot pressures (fore, mid, and rear, kPa) for all participants; * indicates a significant difference (*p* < 0.05) between the data obtained from the two liner groups.

All participants completed the F-Scan gait analysis test at each required study timepoint. Tekscan’s analysis software (v10.0) acquired the ground reaction force (N), stride time (s), and foot pressure (fore, mid, and rear) (kPa) data, which were further calculated and analyzed using a customized MATLAB code. Figure 7 summarizes the analyzed F-Scan average (obtained every 6 weeks) gait test data for all participants. Independent *t*-tests and paired *t*-tests for each participant showed no significant differences in the ground reaction force and stride time. Significant differences were observed for forefoot peak pressure in four participants (participants #7, #11, #13, and #14), midfoot peak pressure in four participants (participants #1, #8, #13, and #14), and rearfoot peak pressure in three participants (participants #7, #13, and #14). In these participants, a trend of higher peak pressure in the three foot regions (seven out of eleven regions) was reported when using conventional liners. Region-specific peak pressure trends could not be established due to the small sample size.

**Figure 8:**
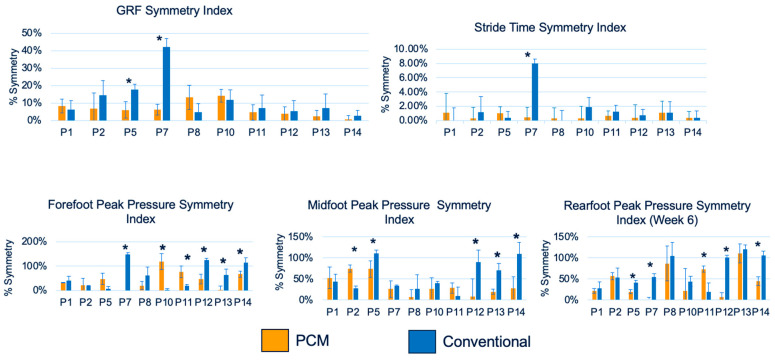
Percentage symmetry (%) for F-Scan data including the ground reaction force (GRF) (N), stride time (s), and foot pressures (fore, mid, and rear, kPa) for all participants at week 6; * *p* < 0.05.

The symmetry index was also calculated for the ground reaction force, stride time, and peak pressures (forefoot, midfoot, and rearfoot) using Equation (1). Complete symmetry between the left and right limbs was indicated by 0%. Figure 8 represents the percent symmetry between the left and right limbs for the ground reaction force (N), stride time (s), and foot pressures (fore, mid, and rear, in kPa) for participants at week 26 for the two liners. The symmetry index was higher in the PCM group for two participants (participants #5 and #7) when comparing the ground reaction force, in one participant (participant #7) for the stride time, in six participants (participants #7, #10, #11, #12, #13, and #14) for the forefoot pressure, in five participants (participants #2, #5, #12, #13, and #14) for the midfoot pressure, and in five participants (participants #5, #7, #11, #12, and #14) for the rearfoot pressure.

## 4. Discussion

Issues with residual limb health are a major concern for prosthesis users [18,19,20]. Prosthetic liners are added mainly to improve suspension, but they also act as a cushioning layer between the residual limb and the prosthetic socket to improve amputees’ safety and comfort. However, the residual limb–liner interface itself provides an environment with many challenges [21]. This dynamic tissue is susceptible to pressure ulcers, rashes, sores, and limb volume changes, depending on the duration of prosthesis use, activity levels, and most importantly the prosthesis’s fit [22,23]. Almost 40–60 percent of individuals are dissatisfied with their prostheses despite the use of liners [24]. While optimizing the prosthetic fit remains the gold standard to mitigate several prosthetic-related problems, excess sweating, due to the poor thermal conductivity of the conventional liners, is an inevitable issue that continues to cause users to wear their prostheses less, thereby affecting their normal active lifestyle and overall wellbeing [9,25,26]. The poor performance of the liners in mitigating the sweat issue can be attributed to the poorly understood in vivo performance of the liner material. There is a need to understand not only the liners’ material properties, but also their functional performance to enhance the prosthetic users’ utilization and performance.

Increased sweating is the primary concern when using conventional liners. It not only leads to acute discomfort but also holds the risk of suspension loss, potentially leading to accidental falls. Furthermore, increased friction due to suspension loss has been reported to be associated with skin damage, thereby prohibiting the user from utilizing the prosthesis and adversely affecting their social participation, employment, and often their psychological health [3,4,5,27]. In recent years, PCM technology has emerged as a promising biosensor technology capable of improving temperature control and, consequently, reducing sweating at the limb–socket interface. A few short-term studies using PCMs have promisingly reported reduced temperature in the liner when compared to conventional liners [11,15].

One of these recent studies investigated the efficacy of built-in cooling channels coupled with a liner with PCMs for keeping an amputee patient’s limb temperature within a narrow range of temperatures. This short-term study recorded the surface temperature of the limb for 60 min and reported the efficacy of PCMs in attaining cooling to be 98.2% alongside built-in cooling channels when compared to silicone liners [15]. In another study, Wernke (2015) had subjects wear SmartTemp liners (WillowWood, Mt. Sterling, OH) and a placebo liner while riding a stationary bike for 25 min, and they measured the residual limb temperature and the amount of sweat inside the liner after the activity. While they directly measured the temperature and the amount of sweat inside the liner after a short biking activity, they did not report performance outcomes such as distance biked or calories burned. While one would expect the findings of less perspiration in PCM liners to result in high performance, the findings from this study did not confirm this hypothesis. In our study, the distance traveled during the 2MWT was used to analyze the users’ performance. While no significant differences were observed in users’ performance as studied using the distance traveled during the 2MWT, the observed lack of any significant differences between the two liners is attributable to the short duration of the 2MWT test. The 2-minute short performance time fails to assess the benefits of PCM liners in long-duration and more intensive tasks [11]. Additional studies are therefore warranted that not only support the efficacy of PCM liners in improving users’ residual limb health by decreasing temperatures within their prosthetic liner, but also report quantitative performance data (recorded over a longer duration) that are critical to support the increase in prosthesis utilization when using PCM liners.

In addition to the short-term studies reporting the efficacy of PCM liners in improving internal socket conditions and user performance, long-term studies are further warranted that support the use of PCM liners by prosthetic users. In our study, we reported the short- and long-term effects of PCM liners on users’ activity levels, performance, and gait biomechanics. Furthermore, we performed a pairwise comparison of the PCM and conventional liners for all of the study parameters, including the step count, 2MWT distance, ground reaction force, stride time, and foot peak pressures in various regions, such as the forefoot, midfoot, and rear foot. Overall, our data reported a trend of better user performance and prosthesis utilization. While the improvements in the average daily step counts and 2MWT distances between the PCM and conventional liner groups were statistically non-significant, five out of the fourteen participants had higher average daily step counts when compared to the conventional liner group. Furthermore, individual participants’ paired *t*-tests for means of biweekly average daily step counts did show significant differences in 2 out of 14 participants. These trends offer some support for the hypothesis that PCM liners lead to an increase in users’ activity levels. This study is unique in reporting the average daily step counts of the same individuals while using PCM and conventional liners. Comparable data in the literature are unavailable, since step counts have been used in prosthesis studies but not for temperature-control liner studies. This study is the first to support direct evidence of trends of increased activity levels and, thus, prosthetic utilization in patients who wear PCM liners.

Another approach used in this study to report the qualitative outcomes of PCM liners was analyzing users’ gait using F-Scan. F-Scan not only offers the ability to assess the gait while using the PCM liners versus the conventional liners, but also helps evaluate symmetry between the normal and prosthetic limbs. Such an approach has been used previously. With the use of the symmetry index, Dingwell et al. (1996) evaluated prosthetic fit, such that a higher symmetry index would indicate improved gait performance [28]. In the current study, notably, foot contact pressure was the only variable that was significantly affected by the liner type. A possible interpretation of this finding is that changes in socket comfort influenced the load-bearing on the prosthetic side. Overall, the F-Scan data reported an increase, with significance in some cases, in the symmetry index in the PCM liner group for the ground reaction force, stride time, and rear foot pressure, thereby supporting the efficacy of PCM technology.

A serious limitation that is likely at the root of the reported lack of significance in the studied parameters is our low sample size. The low sample size of only fourteen participants seriously limited the statistical power needed to confirm the efficacy of PCM technology in this study. This study was part of a multi-institutional study, and the COVID-19 pandemic limited the participant pool to just one of the two possible institutional sites for the 2MWT and the F-Scan gait test. Both study sites did acquire PEQ average scores [29] and step count data, and they reported higher, although non-significant, step counts (PCM: 95%; conventional: 94%) and PEQ average scores for the ambulation (PCM: 75%; conventional: 69%), health (PCM: 71%; conventional: 67%), and utility (PCM: 73%; conventional: 71) sub-scales in the PCM liner group [14]. This study also had a low sample size of twenty-one participants, due to participants dropping out during the pandemic. Future studies are warranted to investigate the utilization and performance outcomes of PCM users while utilizing a larger sample size. Although limited in its scope and design, this study does offer a framework for future studies that are double-blinded, randomized studies with long-term performance outcomes.

In conclusion, this study is unique in reporting the trends of efficacy of PCM liners over conventional liners. The limited participant-specific statistically significant differences found in the studied parameters (step count, 2MWT, ground reaction force, stride time, and foot peak pressure at the fore-, mid-, and rearfoot) support the promising efficacy of the PCM liner over the conventional liner. Providing trends of the obtained quantitative data over previously reported qualitative assessments offers confidence and warrants future studies that investigate the effects of PCM liners when compared to more conventional prosthetic liners. An emphasis on long-term studies is warranted to confirm the efficacy of PCM biosensor technology in mitigating temperature fluctuations within the prosthetic sockets, thereby improving outcomes for people with major limb loss.

## Figures and Tables

**Table 1:** Subject details.

Participant	Age	Gender	Height	Weight
Number	(Year)	(M/F)	(cm)	(kg)
1	66	M	170	86
2	42	M	196	136
5	63	M	178	80
7	36	F	152	64
8	65	M	188	116
10	58	F	178	58
11	61	M	170	122
12	58	M	180	68
13	68	M	175	112
14	67	M	178	100

## Data Availability

Data is available from the corresponding author upon request.
